# Demographic Risk Factors for Suicide among Youths in The Netherlands

**DOI:** 10.3390/ijerph17041182

**Published:** 2020-02-13

**Authors:** Guus Berkelmans, Rob van der Mei, Sandjai Bhulai, Saskia Merelle, Renske Gilissen

**Affiliations:** 1 113Zelfmoordpreventie, Paasheuvelweg 25, 1105 BP Amsterdam, The Netherlands; s.merelle@113.nl (S.M.); r.gilissen@113.nl (R.G.); 2 Centrum Wiskunde & Informatica (CWI), Science Park 123, 1098 XG Amsterdam, The Netherlands; mei@cwi.nl; 3 Vrije Universiteit Amsterdam (VU), De Boelelaan 1085, 1081 HV Amsterdam, The Netherlands; s.bhulai@vu.nl

**Keywords:** suicide, demographics, risk factor, youth

## Abstract

In 2000 to 2016 the highest number of suicides among Dutch youths under 20 in any given year was 58 in 2013. In 2017 this number increased to 81 youth suicides. To get more insight in what types of youths died by suicide, particularly in recent years (2013–2017) we looked at micro-data of Statistics Netherlands and counted suicides among youths till 23, split out along gender, age, regions, immigration background and place in household and compared this to the general population of youths in the Netherlands. We also compared the demographics of young suicide victims to those of suicide victims among the population as a whole. We found higher suicide rates among male youths, older youths, those of Dutch descent and youths living alone. These differences were generally smaller than in the population as a whole. There were also substantial geographical differences between provinces and healthcare regions. The method of suicide is different in youth compared to the population as a whole: relatively more youth suicides by jumping or lying in front of a moving object and relatively less youth suicides by autointoxication or drowning, whereas the most frequent method of suicide among both groups is hanging or suffocation.

## 1. Introduction

Suicide is the number-one cause of death among youths from the age of 10 till the age of 30 in the Netherlands. In July 2018, Statistics Netherlands (CBS) announced that the number of suicides among youths from age 10 up to (not including) 20 had risen to 81 in 2017. In previous years, the number had always been around 50 and below 60: in 2013 there were 58, in 2014 there were 55, and in each of 2015 and 2016 there were 48 suicides among youths from 10 up to 20.

Several risk factors have been identified that lead to youth suicidal behavior, such as previous suicide attempts, feeling hopeless or depressed, alcohol abuse, social isolation and others [1,2,3,4,5]. However, most of these risk factors are psychological and behavioral in nature and thus require a more in-depth look at the individual, and even then, they might be hard to observe. These risk factors are in part derived from psychological autopsy studies where recall bias and a small sample size limit the results [5]. It is useful to know more about the risk of suicide from less in-depth, easier to observe, and more accurately measurable factors such as socio-demographic characteristics. A substantial number of studies into demographic characteristics of suicidal behavior have been done. However, these generally had limited non-random samples and yielded limited results [6]. Also, only a few looked at the demographic characteristics of young suicide victims, and most of these were focused on the United States [7,8]. To get a better understanding of socio-demographic risk factors on youth suicides, the first aim of this study is to look at suicides among all the youths from 10 up till 23 (not including 23) in the Netherlands. The rationale for selecting this age group is that the Dutch government considers this as the youth population for policy purposes. Because we included all the Dutch youths, we have a large dataset without selection bias. We separated out the suicides in the period 2013–2017 by gender, age, residential region, immigration background, place in household and method of suicide and compared them to the corresponding sub-populations of the general population between 10 and 23. Our second aim is to give insight in possible differences in demographic risk factors between youth suicides and suicides in the entire population (including the youths under 23). This could hopefully allow us to find sub-populations among youth suicides that would allow for targeted interventions among youth that would complement interventions targeted among general sub-populations of all ages. A third aim is to see whether there are months or days of the week with a significantly higher amount of suicides among youth and the population as a whole. This could indicate temporal clustering effects and be cause for a further qualitative study.

## 2. Materials and Methods

The data used was micro-data of Statistics Netherlands (CBS) [9]. This data contains information on all inhabitants of the Netherlands (among others: birthdates, municipality they live in (and thus province and Public Health Service region (GGD)), type of household, their role in said household, immigration background, social welfare, and in case of death they include cause of death, date of death, and more) on a yearly basis from various sources which are required to provide this information by law.

Due to the privacy sensitive nature of the data, it is not freely accessible or the data itself allowed to be published. Access has to be granted by Statistics Netherlands on project to project basis, which was granted for this project. It is only possible to work with the data via remote connection to their secure servers and any output is checked by Statistics Netherlands on whether it satisfies the privacy regulations before it is released for publication.

Individuals who died by suicide in the years 2013–2017 were extracted on the basis of their cause of death as established by coroners of the Public Health Services [10] (ICD10 codes for external causes: intentional self-harm (X60–X84)). The coroner is contacted when a person has died and there is any doubt as to whether they have died of natural causes. In the Netherlands, the coroner is always contacted when the deceased is underage, in the Netherlands this means younger than 18 years old. Since the cause of death is provided both privately and anonymously to Statistics Netherlands there is no cause for concern over discrepancies between what the coroner believes the cause of death to be and that which is reported to Statistics Netherlands.

For the reference population (for relative suicide rates and significance checks) we looked at the population at the end of 2017 and included only inhabitants who were listed in the Municipal Personal Records Database, who were at that time 10 years or older (a minimum age standard used because suicide is extremely rare below this age standard in the Netherlands) and who were at that time registered as being a part of a household (all inhabitants of the Netherlands are in both databases and removed upon death or emigration, but occasionally records are not removed from one of the databases due to an administrative error).

For immigration background we use the classification used by Statistics Netherlands. Being of Dutch descent means having both Dutch parents. If exactly one of the parents is an immigrant, we say the youth has an immigration background corresponding to the country of origin of said parent. If both parents are immigrants, we consider only the country of origin of the mother. Lastly, if the youth is an immigrant themselves we say they have an immigration background corresponding to the country of origin. Countries classified as western are countries from Europe (Turkey excluded), North America, Oceania, and the countries Indonesia and Japan. Countries classified as non-western are countries from South America, Africa, and Asia (Indonesia and Japan excluded) and additionally Turkey.

For tests of significant differences between sub-populations we used the chi-square test of homogeneity with a significance level of 0.05. We compared the frequencies of the sub-population within the suicide victims to the frequencies of the sub-population within the corresponding reference population. In the case where significant differences were found to be present we subsequently looked at residuals and used thresholds of −2 for significantly lower and 2 for significantly higher. We did not correct for multiple comparisons since this is not desirable in an explorative study [11].

## 3. Results and Discussion

### 3.1. Disclaimer

Due to privacy concerns, numbers strictly lower than 10 could not be reported. In addition, to prevent those numbers to be able to be deduced from the remaining numbers, some other numbers also had to be hidden. All hidden numbers have been replaced by * in the tables. They are still taken into account when doing tests of significance; however chi-squared values, residuals and *p*-values have not been reported since it might be possible to deduce some of the hidden numbers from these values.

### 3.2. Gender

From the data, we observe that yearly among youths under 23 roughly 1.5 to 2 times as many males than females died by suicide in the period 2013–2017 (Table 1), 331 male youths and 170 female youths, with the number of males varying more than the number of females. When compared to the entire population (Table 2), we observe that this ratio is even higher: males consistently died by suicide more than twice as often as females with 6421 male suicide victims and 2956 female suicide victims during the entire period.

### 3.3. Age

Looking at the age of the suicide victims under 23 (Table 3), we observe that older youths are more likely to die by suicide with the number of suicides increasing until we get to 19 years old with 77 suicides, 73 suicides at 20 years old, 76 suicides at 21 years old and 74 suicides at 22 years old during the period 2013–2017. There was no statistically significant difference in the number of suicides among youths under 23 in the years in the study period.

### 3.4. Province and Healthcare Regions

Looking at provinces (Table 4), we see substantial differences with the highest provincial suicide rates among youths in Groningen and Noord-Brabant with 5.47 and 5.15 per 100,000 youths per year, respectively. This is more than twice than that of the lowest provincial suicide rate: Zuid-Holland with 2.50 per 100,000 per year. The provinces Groningen, Noord-Brabant and Gelderland had significantly higher suicide rates among youths than the rest of the country, whereas Zuid-Holland had significantly lower suicide rates among youths than the rest of the country. When looking at the whole population, the provinces Groningen, Noord-Brabant, Friesland, Drenthe and Limburg have significantly higher suicide rates than the rest of the country while Overijssel, Utrecht and Zuid-Holland have significantly lower suicide rates.

Among so-called Municipal Health Service Regions (regions where municipalities organize healthcare together, also known as GGD regions) even larger differences can be observed with the lowest observed rate of suicides for youths being 2.14 per 100,000 and the highest 5.73 per 100,000 (Table 5). The lowest observed rate for the population as a whole is 8.32 per 100,000 in South Holland South and the highest being Groningen with 13.92 per 100,000. However, this is to be expected due to the fact that we are dealing with more regions and even smaller population sizes, which causes the variability on the relative suicide rates to increase. We see that generally high suicide rates among youths coincide with high suicide rates among the population as a whole. What is interesting to note is that both the suicide rates of youths and the suicide rates of the population as a whole are relatively low in the Municipal Health Service Regions containing the four largest cities of the Netherlands: Amsterdam, Rotterdam, Utrecht and the Hague (collectively known as the “Randstad”). The GGD regions with significantly high suicide rates among youths are GGD Groningen, Security and Health Region (SHR) Middle Gelderland, GGD North Holland North, GGD Heart for Brabant and GGD Brabant Southeast and the ones with significantly low suicide rates are GGD Amsterdam, GGD Rotterdam- Rijnmond, and Health and Youth Service (HYS) South Holland South. The GGD regions with significantly high suicide rates among the whole population are GGD Groningen, GGD Drenthe, GGD West-Brabant, GGD Heart for Brabant, GGD Limburg South and GGD Fryslân and the ones with significantly low suicide rates among the entire population are GGD Region Twente, GGD Region Utrecht, GGD Kennemerland, GGD Hollands-Midden, GGD Rotterdam-Rijnmond, HYS South Holland South, GGD Haaglanden.

When considering regions it is important to note that suicide rates among in-patients of psychiatric institutions are many times higher than the average suicide rates [12] and these institutions are not spread homogeneously across the country, so high regional suicide rates could be due to the in-patients of said institutions. Also, the effect possible suicide clusters might have will also affect the suicide rate heavily (since the number of suicides in most regions are relatively small).

### 3.5. Immigration Background

When looking at the immigration background of the youths who died by suicide (Table 6), we observe that 75% were of Dutch descent, 10% had a western immigration background and 15% had a non-western immigration background. The suicide ratio among those of a non-western immigration background was significantly lower than the average suicide ratio in the youth population as a whole. However, neither the suicide rate among youths of Dutch descent nor the suicide rate among youths with a western immigration background can be shown to be significantly higher than the suicide rate among all youths. When considering the entire population (Table 7), we observe that not only is the suicide rate among people with a non-western immigration background significantly lower, the suicide rate among people of Dutch descent and the suicide rate among people with a western migration background are both significantly higher than the population as a whole which is consistent with findings in Belgium [13]. The fact that non-western immigrant youth had lower suicide rates than other youth was consistent with findings from Ontario and Switzerland [14,15]. In addition, although we only had data on fatal attempts it has been previously reported that young female non-western immigrants were more likely to attempt suicide [16].

### 3.6. Place in Household

When considering the place the youths occupy within a household, we observe that youths living with their parents are significantly less likely to die by suicide than youths not living with their parents (Table 8). Although they make up over 60% of youth suicides, they make up a larger proportion of the youth population as an entirety. Within the group of youths not living with their parents, we observe that youths living on their own are significantly more likely to die by suicide. The group least likely to die by suicide are non-married youths living with their partner who do not have any children.

### 3.7. Method of Suicide

Among youths who die by suicide, we see that the most common method of suicide (47%) is strangulation or suffocation (which includes hanging), followed by jumping or lying in front of a moving object (most often railway suicides; 33%) (Table 9). In the general population, strangulation and suffocation is also responsible for 47% of suicide deaths (Table 10). However, jumping or lying in front of a moving object is responsible for 11% of suicide deaths which is substantially lower than the 33% among youths. We see that 21% of deaths among the general population is due to self-poisoning (this includes drugs, both medicinal and recreational, alcohol, gas, bleach and others), whereas among youths it accounts for 8% of suicide deaths. The disparity between methods is possibly in part due to the fact that adults are more likely to have access to the means required for autointoxication. This could also explain the high rates among youths for jumping or lying in front of moving objects since the rail is relatively easily accessible and does not require any other means. The fact drowning is a more common method of suicide for adults seems to be consistent with a Norway study which found that drowning was mostly used by older women [17].

### 3.8. Month and Day of the Week

If we look at how suicides were distributed among youths in the various months in the period 2013–2017, none of the months have a significantly high or low amount of suicides (Table 11). Among youth in the United States a significant increase was found in the amount of suicides in March and April of 2017, which was associated with the release of the Netflix series “13 Reasons Why” [18]. However, no such increase was found in suicides among the youth in the Netherlands in those same months.

The amount of suicides among youth varies across days of the week (Table 12); however, there is no statistically significant difference. This is noteworthy since in Ireland a significant difference was found in which days of the week young people died by suicide [3]. They saw that suicide was concentrated in the period from Saturday till Monday and theorized that this could be due to increased alcohol consumption in the weekend. The fact that Dutch youths tend to drink mostly on Friday and Saturday [19] which have the lowest rates of suicide (although not statistically significant) does not provide additional evidence for a clear relation between alcohol abuse and youth suicide in the Netherlands. This is different among the whole population; we do see a significant difference in the suicide rates throughout the days of the week (Table 13). Also note that the lower amount of suicides on Saturdays is consistent throughout the examined period. The difference in distribution of the youths and the Dutch population as a whole is not significant, so it cannot be concluded that there are differences in distribution of weekdays between youths and the population as a whole. The fact Monday shows a significantly higher amount of suicides among the whole population is consistent with recent studies in the UK, Australia and Korea [20,21,22].

### 3.9. Limitations and Strengths

When interpreting the results it is important to note that even though we observe various statistical differences between the various sub-populations obtained from our socio-demographic characteristics, the individual effects of said characteristics are harder to measure due to the heavily correlated nature of the characteristics. The youths under 18, for example, are way more likely to live with their parents than to live on their own compared to the youths older than 18, so it becomes difficult to measure whether or not the suicide rate is higher among youths who live alone due to an isolation factor or due to the fact that these youths are usually the older ones. Similarly, the various geographical regions will have a different demographic makeup thus making it hard to separate out the various effects. We also do not know how the various effects interact and stack. In addition, due to privacy concerns the amount of suicides in some sub-populations could not be reported leading to an incomplete view. However, these unreported values were taken into account for tests of significance. Also totals over the entire period 2013–2017 could often be reported so the impact of not being able to report these specific values was limited. A major strength is the quality and quantity of the data. The CBS has data on everyone in the Netherlands and only makes datasets available when their quality has been thoroughly checked. Some datasets are also systematically updated once or twice in case new information has become known. This results in datasets of high quality.

### 3.10. Generalizability

The results agreed with some results from earlier studies done in other populations in some respects such as immigration background [13,14,15], suicide being more common on Mondays [20,21,22], and drowning being more common among adults. On the other hand there are also some results that contrast with studies done in other populations, such as not having more youth suicides in the weekend [3] or no increase in suicides in the period surrounding the release of 13 Reasons Why [18]. This suggests some results might be generalizable to other countries whereas some others are not, due to possible cultural elements.

## 4. Conclusions

We have managed to obtain unbiased frequencies of suicide in various sub-populations of both youths and the population as a whole. This showed us that there was a higher risk of suicide among older youths, male youths, youths living alone, those of Dutch descent and those living in certain regions (Groningen etc.). The lowest risks are seen among youths who live with their parents, younger youths, female youths and youths living in or around the largest cities in the Netherlands.

The most common method of suicide among both young suicide victims and adults were strangulation or suffocation. The second most common was jumping or lying in front of moving objects for youths but self-poisoning for adults. We do not see any significant changes in causes of death. However, this could be due to the period only being 5 years as trends might occur slowly over a longer period of time [17]. There was no significant difference in the number of youth suicides among months or days of the week. In the population as a whole however we do see significant differences in days of the week with a peak at Monday and a trough at Saturday.

We found that the main differences between the risk factors of youth and the general population is one of effect size. Males have higher risk than females and this effect is greater in the general population than in the youth population. Similarly, the protective factor of being a non-western immigrant is larger in the general population than in the youth population. This suggests that these effects accumulate as one ages, for example through continued exposure to certain expectations or to a certain culture. Sadly, this makes focusing on demographic risk groups for youth less effective than it would be for adults.

We also found differences in methods, youths die more often due to jumping or lying in front of moving objects whereas adults die more often to self-intoxication or drowning. Restricting access to means for hanging or strangulation is hardly feasible unless the individual is restricted to a closed institution. Therefore, the best restriction to means for youths would be to focus on hotspots for railway suicides.

In future research we intend to look at decorrelating effects and examine the way various effects interact, and whether there are combinations of risk factors that are cause an especially high risk of suicide. We also intend to investigate whether or not changes in risk factors throughout time result in a substantial change in suicide risk and, if so, what kind of changes these are. 

## Figures and Tables

**Table 1:** Number of male and female suicides among Dutch youths under 23 years old in the years 2013 to 2017 (percentage of total in year in brackets).

Year	Male	Female	Total
2013	73 (66%)	38 (34%)	111
2014	56 (59%)	39 (41%)	95
2015	65 (68%)	30 (32%)	95
2016	59 (63%)	34 (37%)	93
2017	78 (67%)	39 (33%)	117

**Table 2:** Number of male and female suicides among the entire Dutch population in the years 2013 to 2017 (percentage of total in year in brackets).

Year	Male	Female	Total
2013	1308 (74%)	549 (26%)	1857
2014	1250 (68%)	589 (32%)	1839
2015	1280 (68%)	591 (32%)	1871
2016	1279 (68%)	614 (32%)	1893
2017	1304 (68%)	613 (32%)	1917

**Table 3:** Number of suicides by age of youths under 23 in the period 2013–2017 (percentage of total under 23 in brackets).

Age	Number of Suicides
10–13	19 (4%)
14	17 (3%)
15	24 (5%)
16	37 (7%)
17	50 (10%)
18	64 (13%)
19	77 (15%)
20	73 (14%)
21	76 (15%)
22	74 (14%)

**Table 4:** Number of suicide victims under 23 in the period 2013–2017 by province (RS = Relative Suicide Rate per 100,000 per year) (percentage of total in Netherlands in brackets).

Province	Suicides Youths (N)	RS Youths	Suicides Whole Pop. (N)	RS Whole Pop.
Netherlands	511 (100%)	3.86	9377 (100%)	12.27
Groningen	25 (5%)	5.47	346 (4%)	13.92
Friesland	16 (3%)	3.21	396 (4%)	13.18
Drenthe	14 (3%)	3.60	314 (3%)	12.76
Overijssel	28 (5%)	2.83	569 (6%)	9.89
Flevoland	14 (3%)	3.64	199 (2%)	9.67
Gelderland	78 (15%)	4.51	1143 (12%)	11.10
Utrecht	32 (6%)	2.93	606 (6%)	9.36
Noord-Holland	76 (15%)	3.32	1491 (16%)	10.54
Zuid-Holland	78 (15%)	2.50	1745 (19%)	9.34
Zeeland	*	*	227 (2%)	11.88
Noord-Brabant	106 (21%)	5.15	1547 (16%)	12.45
Limburg	31 (6%)	3.61	669 (7%)	11.97

**Table 5:** Number of suicide victims under 23 and among the population as a whole in the period 2013–2017 by Municipal Health Service (GGD) region (RS = Relative Suicide rate per 100,000 per year, SHR = Security and Health Region, HYS = Health and Youth Service) (percentage of total in Netherlands in brackets).

GGD Region	Suicides Youths	RS Youths	Suicides Whole Pop.	RS Whole Pop.
GGD Groningen	25 (5%)	5.47	346 (4%)	13.92
GGD Fryslân	16 (3%)	3.21	396 (4%)	13.18
GGD Drenthe	14 (3%)	3.60	314 (3%)	12.76
GGD Ijsselland	14 (3%)	3.14	274 (3%)	10.47
GGD Region Twente	14 (3%)	2.57	295 (3%)	9.40
GGD North- and East-Gelderland	26 (5%)	3.93	443 (5%)	10.79
SHR Middle Gelderland	32 (6%)	5.46	369 (4%)	10.77
GGD Gelderland South	20 (4%)	4.14	331 (4%)	11.96
GGD Flevoland	14 (3%)	3.64	199 (2%)	9.67
GGD Region Utrecht	32 (6%)	2.93	606 (6%)	9.36
GGD North Holland North	30 (6%)	5.73	373 (4%)	11.39
GGD Kennemerland	13 (3%)	2.99	239 (3%)	8.83
GGD Amsterdam	20 (4%)	2.32	541 (6%)	10.34
GGD Gooi en Vechtstreek	*	*	152 (2%)	12.02
GGD Middle Holland	23 (5%)	3.43	370 (4%)	9.32
GGD Rotterdam-Rijnmond	23 (5%)	2.14	626 (7%)	9.61
HYS South Holland South	*	*	204 (2%)	8.32
GGD Zeeland	*	*	227 (2%)	11.88
GGD West-Brabant	25 (5%)	4.44	456 (5%)	12.98
GGD Heart for Brabant	46 (9%)	5.30	669 (7%)	12.64
GGD Brabant SouthEast	35 (7%)	5.59	449 (5%)	11.71
GGD Limburg North	18 (4%)	4.59	297 (3%)	11.46
GGD Limburg South	13 (3%)	2.79	372 (4%)	12.42
GGD Haaglanden	25 (5%)	2.59	545 (6%)	9.97
GGD Zaanstreek/Waterland	*	*	186 (2%)	11.11

**Table 6:** Number of suicides among youths under 23 of Dutch descent, youths with a western migration background and youths with a non-western immigration background (percentage of total in year in brackets).

Year	Dutch Descent	Western Immigration Background	Non-Western Immigration Background
2013	89 (80%)	*	*
2014	70 (74%)	12 (13%)	13 (14%)
2015	68 (72%)	*	*
2016	70 (75%)	*	*
2017	86 (74%)	11 (9%)	20 (17%)
Total	383 (75%)	52 (10%)	76 (15%)

**Table 7:** Number of suicides among people of Dutch descent, people with a western migration background and people with a non-western immigration background among the entire population (percentage of total in year in brackets).

Year	Dutch Descent	Western Immigration Background	Non-Western Immigration Background
2013	1570 (85%)	162 (9%)	114 (6%)
2014	1507 (82%)	216 (12%)	104 (6%)
2015	1539 (82%)	206 (11%)	117 (6%)
2016	1536 (81%)	232 (12%)	112 (6%)
2017	1561 (81%)	207 (11%)	142 (7%)

**Table 8:** Number of suicides among youths under 23 separated out by place in household (percentage of total in year in brackets).

Year	Living with Parents	Living Alone	Partner Non-Married Couple without Children	Member of Institutional Household	Other
2013	82 (74%)	20 (18%)	*	*	*
2014	64 (67%)	20 (21%)	*	*	*
2015	65 (68%)	19 (20%)	*	*	*
2016	63 (68%)	23 (25%)	*	*	*
2017	83 (71%)	26 (22%)	*	*	*
Total	357 (70%)	108 (21%)	13 (3%)	23 (5%)	10 (2%)

**Table 9:** Number of suicides among youths under 23 separated out by method of suicide (percentage of total in year in brackets).

Year	Self-Poisoning	Strangulation or Suffocation	Drowning	Jumping from High Place	Jumping or Lying in Front of Moving Object	Other
2013	*	49 (44%)	*	*	38 (34%)	*
2014	*	46 (48%)	*	*	29 (31%)	*
2015	*	39 (41%)	*	*	32 (34%)	*
2016	*	42 (45%)	*	*	36 (39%)	*
2017	*	65 (56%)	*	*	35 (30%)	*
Total	41 (8%)	241 (47%)	10 (2%)	36 (7%)	170 (33%)	13 (3%)

**Table 10:** Number of suicides among the general Dutch population separated out by method of suicide (percentage of total in year in brackets).

Year	Self-Poisoning	Strangulation or Suffocation	Drowning	Jumping from High Place	Jumping or Lying in Front of Moving Object	Other
2013	345 (19%)	926 (50%)	105 (6%)	136 (7%)	201 (11%)	144 (8%)
2014	397 (22%)	877 (48%)	111 (6%)	138 (8%)	188 (10%)	128 (7%)
2015	432 (23%)	859 (46%)	111 (6%)	123 (7%)	212 (11%)	134 (7%)
2016	428 (23%)	864 (46%)	111 (6%)	147 (8%)	220 (12%)	123 (6%)
2017	401 (21%)	909 (47%)	116 (6%)	136 (7%)	219 (11%)	136 (7%)
Total	2003 (21%)	4435 (47%)	554 (6%)	680 (7%)	1040 (11%)	665 (7%)

**Table 11:** Number of suicides a month among youths under 23 in the period 2013–2017.

Year	Jan.	Feb.	March	April	May	June	July	Aug.	Sep.	Oct.	Nov.	Dec.
2013	10	*	13	13	11	14	*	*	*	*	*	*
2014	*	*	13	*	*	*	*	*	*	*	12	*
2015	*	*	*	*	11	*	*	*	12	11	*	*
2016	10	*	*	*	*	*	*	*	*	14	11	*
2017	11	12	*	*	10	12	13	*	*	11	13	14

**Table 12:** Number of suicides among the Dutch population under 23 for each day of the week over the period 2013–2017.

Year	Sunday	Monday	Tuesday	Wednesday	Thursday	Friday	Saturday
2013	21	19	14	16	19	*	*
2014	13	11	16	12	16	10	17
2015	14	11	16	15	13	16	10
2016	13	15	16	20	13	*	*
2017	11	19	17	20	20	19	11
Total	72	75	79	83	81	67	54

**Table 13:** Number of suicides among the general Dutch population for each day of the week over the period 2013–2017.

Year	Sunday	Monday	Tuesday	Wednesday	Thursday	Friday	Saturday
2013	243	317	291	278	265	266	197
2014	255	288	283	261	270	246	236
2015	236	295	303	311	264	260	202
2016	222	311	315	280	269	280	216
2017	254	353	292	278	264	254	222
Total	1210	1564	1484	1408	1332	1306	1073
